# Right Hemisphere Remapping of Naming Functions Depends on Lesion Size and Location in Poststroke Aphasia

**DOI:** 10.1155/2017/8740353

**Published:** 2017-01-12

**Authors:** Laura M. Skipper-Kallal, Elizabeth H. Lacey, Shihui Xing, Peter E. Turkeltaub

**Affiliations:** ^1^Department of Neurology, Georgetown University Medical Center, Washington, DC, USA; ^2^Research Division, MedStar National Rehabilitation Hospital, Washington, DC, USA; ^3^Department of Neurology, First Affiliated Hospital of Sun Yat-Sen University, Guangzhou, China

## Abstract

The study of language network plasticity following left hemisphere stroke is foundational to the understanding of aphasia recovery and neural plasticity in general. Damage in different language nodes may influence whether local plasticity is possible and whether right hemisphere recruitment is beneficial. However, the relationships of both lesion size and location to patterns of remapping are poorly understood. In the context of a picture naming fMRI task, we tested whether lesion size and location relate to activity in surviving left hemisphere language nodes, as well as homotopic activity in the right hemisphere during covert name retrieval and overt name production. We found that lesion size was positively associated with greater right hemisphere activity during both phases of naming, a pattern that has frequently been suggested but has not previously been clearly demonstrated. During overt naming, lesions in the inferior frontal gyrus led to deactivation of contralateral frontal areas, while lesions in motor cortex led to increased right motor cortex activity. Furthermore, increased right motor activity related to better naming performance only when left motor cortex was lesioned, suggesting compensatory takeover of speech or language function by the homotopic node. These findings demonstrate that reorganization of language function, and the degree to which reorganization facilitates aphasia recovery, is dependent on the size and site of the lesion.

## 1. Introduction

One-third of stroke survivors suffer from loss of language ability [1, 2]. Recovery rates vary greatly, for reasons that are poorly understood [3]. The relationships between lesion site, activity pattern changes, and recovered language functions remain unclear.

### 1.1. Reorganization of Language Function after Stroke

In neuroimaging studies, patterns of increased activation during language tasks in chronic aphasia have been broadly consistent across studies. In a meta-analysis of neuroimaging studies, collapsing across a wide range of language tasks, we found that people with aphasia consistently overactivated perilesional regions in the left hemisphere, as well as right hemisphere regions that were homotopic to the left hemisphere language network [4]. In particular, people with lesions in the left inferior frontal gyrus (IFG) were more likely to recruit right IFG than those without lesions in that area. However, the behavioral and biological drivers of these changes, as well as the degree to which they promote, inhibit, or are even relevant to recovery, remain open questions.

The increased activation in the preserved left hemisphere in people with aphasia has generally been associated with overall better performance [5–8]. However, the relationship between lesion size, location, and ability to use these preserved regions has not been carefully examined. For example, particularly severe participants may have larger lesions or lesions in highly critical areas. If this is the case, then the relationship between left hemisphere activity and language performance may be indirect, in that both are actually dependent on the severity and size of the stroke itself, as well as the availability of left hemisphere tissue adjacent to the critical areas.

Within the right hemisphere, the relationship between plasticity and language performance is even less clear. A number of lines of evidence suggest that engagement of the right hemisphere serves overall to support aphasia recovery (for review, see [9]), suggesting a compensatory role for the right hemisphere homologues to language nodes. However, transcranial magnetic stimulation (TMS) studies show that inhibiting the right IFG pars triangularis (PTr) improves fluency, naming, and other language measures in people with left hemisphere stroke [10–15]. This suggests that the right IFG, specifically right PTr, may be limiting recovery in people with left hemisphere lesions. Furthermore, neuroimaging studies show that early engagement of the right hemisphere during the acute phase promotes recovery but that disengagement of the right hemisphere in later stages is related to ongoing successful recovery [16–19]. Increased activation in the right hemisphere during the chronic stage of aphasia is associated with naming errors [20] and overall worse performance, especially in picture-word naming and rhyme judgment [16, 21].

Unfortunately, the same concerns arise when examining the function of the right hemisphere as were raised for the left, specifically, confounding with lesion size and location. The usefulness of a shift from right to left hemisphere activation during the chronic stages likely depends on the availability of remaining healthy left hemisphere tissue [5]. Additionally, right hemisphere recruitment identified in neuroimaging studies may not be a consequence of plasticity at all. In neurologically healthy control subjects, right hemisphere activation has been shown to increase as a function of task difficulty [22, 23]. Furthermore, right hemisphere activity appears to be greater in participants with larger overall left hemisphere lesions [24], although this finding is only for some language tasks (picture naming, but not semantic judgment). Right hemisphere activity in people with aphasia, therefore, may actually be driven by the unique difficulty of the language tasks for people with aphasia. Right hemisphere activity may also reflect overactivation of any preserved tissue, of which there is less for people with large lesions, rather than actually remapping of function. However, the relationship between activity in the right hemisphere in people with aphasia, the rate of recovery, and actual plasticity remains unclear.

Beyond the general anatomical patterns of reorganization and their association with good or bad outcomes, the mechanisms underlying reorganization remain unclear. Such mechanisms may include behaviorally driven reorganization, as in the plasticity induced by speech-language therapy [18], or direct biological effects of the stroke itself. With regard to direct biological mechanisms underlying plasticity in language networks, relatively few specific hypotheses have been put forth. While some investigators, including ourselves, have previously described different patterns of reorganization (e.g., compensatory “takeover” by a new area), these descriptions do not generally hypothesize a specific biological basis for these changes. The great virtue of specific biological hypotheses is that they generate specific testable predictions, especially with regard to the timing of plasticity, the relationship of specific lesion features such as size and location to the pattern of reorganization, and the relationship of brain changes to behavioral outcomes.

One such biological hypothesis which has gained a great deal of traction in recent years is the interhemispheric inhibition model, which is commonly invoked to explain recruitment of homotopic right hemisphere processors, negative relationships between right hemisphere activity and language performance, and the beneficial effects of right PTr inhibition [25]. This hypothesis states that, in healthy people, there is transcallosal cross-hemispheric inhibition between language areas, similar to what has been demonstrated in motor areas [26, 27]. A stroke in the left hemisphere theoretically disrupts the interhemispheric inhibitory balance, leading to overactivation of right hemispheric language areas homotopic to the lesion. In the context of the interhemispheric inhibition theory, the overactivated right hemisphere is thought to maladaptively inhibit perilesional left hemisphere areas, resulting in worse outcomes [14].

In particular, the interhemispheric inhibition model makes at least three specific testable claims. First, larger lesions in the left hemisphere should be associated with more activity in the right hemisphere. Second, left hemisphere lesions should be associated with increased activity specifically in homotopic regions in the right hemisphere. Finally, increased right hemisphere activity in homotopic areas should be related to worse language abilities, even at the chronic stage. Some of the prior evidence regarding the latter two claims is outlined above. The predicted relationship between lesion size and right hemisphere recruitment is frequently mentioned in reviews on aphasia recovery either in the context of interhemispheric inhibition or based on the logical argument that if no left hemisphere tissue remains, the right hemisphere must be recruited for any recovery to occur [7, 28, 29]. However, the empirical evidence supporting this relationship is lacking and based primarily on small case series using methods that do not control for the accuracy of task performance [30].

### 1.2. Cognitive Models of Naming

Lexical-retrieval processes involve accessing concept knowledge and mapping phonological representations, stored in long-term memory [31] and these two stages are supported by distinct cortical regions. Phonological retrieval is ascribed to a ventral stream of processing, in which phonological representations in the posterior superior temporal lobe map onto semantic and conceptual information in the angular gyrus and anterior temporal lobes [32].

Postlexical output is the production stage, in which phonological representations are mapped to motor representations and speech occurs. Some dual-stream models assign this stage to the dorsal stream, in that the phonological representations in the superior temporal gyrus (STG) are mapped to motor sequence representations in the temporoparietal junction and posterior inferior frontal lobes [32].

### 1.3. Design of This Experiment

We tested these three predictions of the interhemispheric inhibition model in a cross-sectional study of people with chronic aphasia following left hemisphere stroke. The experiment used an fMRI task to isolate covert (phonological retrieval) and overt (postlexical output) phases of picture naming; we first examined the general patterns of activity in our aphasia group compared to matched controls and the relationship between activity and overall lesion size. We then used the activity in the control group to define the normal brain network for naming in older people without language impairment or brain injury and tested how lesions at key nodes in this network affected activity throughout the rest of the brain, in particular within the remaining bilateral language sites. The goal of the study was to examine whether plastic changes in chronic aphasia would be related to the size and site of the lesion, with particular interest in right hemisphere plasticity occurring in regions that were homotopic to the left hemisphere lesions. We further tested whether any changes in activity associated with lesion site were related to naming performance, in order to assess whether these regions are successfully adapting to support language function or are inhibiting successful recovery.

## 2. Materials and Methods

### 2.1. Participants

Forty-nine chronic left hemisphere stroke survivors with a history of aphasia were recruited. Ten participants were then excluded based on fMRI task performance (<10% accuracy) resulting in a final sample of 39 participants in the aphasia group.

All participants in the aphasia group were native English speakers and testing occurred at least six months after their stroke (mean chronicity = 52.9 months). Participants were screened based on ability to follow testing instructions and had no history of other significant neurological illnesses. The distribution of lesions and individual demographic information for the aphasia group can be found in the Supplementary Material available online at https://doi.org/10.1155/2017/8740353.

Thirty-seven healthy control subjects, with no neurologic or psychiatric disorders, were also tested. Participants in the control group were matched to the aphasia group on age (*t*(67.5) = −0.42, *P* > 0.60), sex (*χ*^2^(1) = 0.79, *P* > 0.30), education (*t*(73.0) = 0.85, *P* > 0.30), and handedness (*χ*^2^(3) = 3.1, *P* > 0.30). Group means can be found in Table 1.

The study was approved by the Georgetown University Institutional Review Board, and written informed consent was obtained from all study participants prior to enrollment in the study.

### 2.2. Experimental Design

Visual stimuli consisted of 54 line drawings, with 92–100% name agreement based on norming in an independent sample of 55 older controls, representing one-, two-, and three-syllable words. To reduce individual differences in in-scanner performance, participants were presented with one of two 32-item sets during scanning based on the severity of their deficits. Fourteen participants whose naming and repetition deficits were severe in pre-MRI testing were given 32 one- and two-syllable items, while all other participants, including controls, were given 32 two- and three-syllable items during scanning. The one-syllable words also had overall higher frequency than the three-syllable words.

The fMRI task followed a slow jittered event related design. The trials were presented in a pseudo-randomized order. The task was a delayed naming task, which allows for the independent analysis of name retrieval and name production [33]. First, a single line drawing appeared centered on the screen, surrounded by a red border. This image remained on the screen for 7500–9000 ms, during which time the participant named the object in the image silently (covert naming). Then, the border around the image changed from red to green and remained on screen for 5500 ms. During this time, the participant was asked to produce the name of the object aloud (overt naming). Finally, the line drawing and the surrounding box disappeared and the participant fixated on a crosshair for 14000 ms. A slow event related design was chosen to allow for wash out of the hemodynamic response, which may be slower in stroke survivors [34]. Images were presented using E-Prime software (Psychology Software Tools Inc., Pittsburg, PA), and responses were recorded using a MRI safe microphone (Opto-acoustics, FOMRI-III). Before the scan, participants practiced the task on images not included in the fMRI task. If a participant produced the correct name at any point during the overt naming period, the item was counted as correct. Only trials in which the correct response was produced during the overt phase were included in the analysis. If a participant made an incorrect or no response during the overt naming phase of the trial, the entire trial (both covert and overt phases) was removed from further analysis.

Naming ability was tested using a 60-item version of the Philadelphia Naming Test (PNT) [35], made up of items independent of those used in the scanning task. Testing took place within one week of the MRI scan. We counted the total number of items on the PNT that were named correctly on the first attempt.

### 2.3. Scanning Parameters and Preprocessing

MRI data were collected on a 3.0 T Siemens Trio Scanner at the Georgetown University Medical Center. A high resolution T1-weighted MPRAGE was collected with the following parameters: repetition time = 1900 ms, echo time = 2.56 ms, flip angle = 9°, 160 contiguous 1 mm slices, field of view = 250 × 250 mm, matrix size = 246 × 256, and voxel size = 1 × 1 × 1 mm. Functional T2^*∗*^-weighted images were acquired using a gradient-echo echo-planar pulse sequence, with the following parameters: repetition time = 2000 ms, echo time = 30 ms, flip angle = 90°, 38 contiguous 3.2 mm slices, field of view = 250 × 250 mm, and voxel size = 3.2 × 3.2 × 3.2 mm. The functional scan consisted of 32 trials, including an opening and closing screen, totaling approximately 15 minutes.

Lesion masks were created by manually tracing stroke damage on the T1-weighted images, in native space, in MRIcron [36], following a preestablished set of guidelines for determining lesion borders. Ventricular expansion was not included in the lesion. All lesion masks were checked by two board certified neurologists (Shihui Xing and Peter E. Turkeltaub) after the tracing and again after the lesion masks were warped to the template.

fMRI data were preprocessed and analyzed using FSL 5.0.6 [37]. Preprocessing included application of a high pass temporal filter, standard correction for head motion using MCFLIRT, interleaved slice timing correction, intensity normalization across volumes, and spatial smoothing to 5 mm FWHM. Registration and normalization were carried out to the MNI standardized brain provided by FSL. For each condition in each trial, a canonical double-gamma hemodynamic response function was constructed for the duration of the event. Motion parameters were then included as covariates in the model.

### 2.4. fMRI Analysis

First, we examined where participants in the aphasia group over- and underactivated relative to controls during covert and overt naming. In between-group contrasts, only areas that were significantly active (*z* > 2.3) in the aphasia group were included in the aphasia > control contrast, and vice versa. All whole-brain analyses were examined at cluster corrected *P* < 0.01, after a grey matter mask was applied.

#### 2.4.1. Effects of Lesion Volume on Activity

We tested whether right hemisphere activity in people with aphasia is driven by the extent of overall damage in the left hemisphere. To do this, we quantified the size of the lesion, warped to template, for each individual. Lesion size was measured in mm^3^ after warping to a standardized template and then entered as a voxelwise continuous predictor variable in a group analysis. Clusters identified as significant in this analysis are areas where activation, for either covert or overt naming, differed as a function of lesion size.

#### 2.4.2. Regions of Interest Used to Examine Remapping

In order to examine how lesions within the normal language network affect naming ability, we identified regions of interest (ROIs) using the within-group contrasts from the control group. The peak voxel in each active cluster was identified, excluding primary visual cortex, for both the covert > fixation and overt > fixation contrasts. Then, 5 mm spheres were drawn around the peak voxel.

For each of the left hemisphere ROIs, aphasia group participants were grouped based on lesion status at the ROI. As these were very small ROIs, not large clusters, the distributions of percent lesions in the ROIs were highly bimodal, so an all or nothing approach was used: If a participant had a lesion that overlapped with the ROI in even one voxel, the participant was counted in the “lesion” group for that ROI. Then we tested whether lesions at each left hemisphere site led to worse naming performance on the PNT. Whole-brain analyses were then carried out, contrasting activity in these two groups (lesion versus intact at ROI site) of people with aphasia, while controlling for overall lesion volume.

Finally, we examined more closely the relationships between damage in sites normally active in controls and activity in the remaining nodes in the network. Regressions were carried out which tested whether lesion status in a left hemisphere ROI predicted activity levels in each of the other left and right hemisphere ROIs, controlling for lesion volume. ROIs in which activity was modulated by lesion status at another site were then further tested to see whether activity in that area related to naming ability.

## 3. Results

### 3.1. Whole-Brain Activity during Covert and Overt Naming

**Figure 1:**
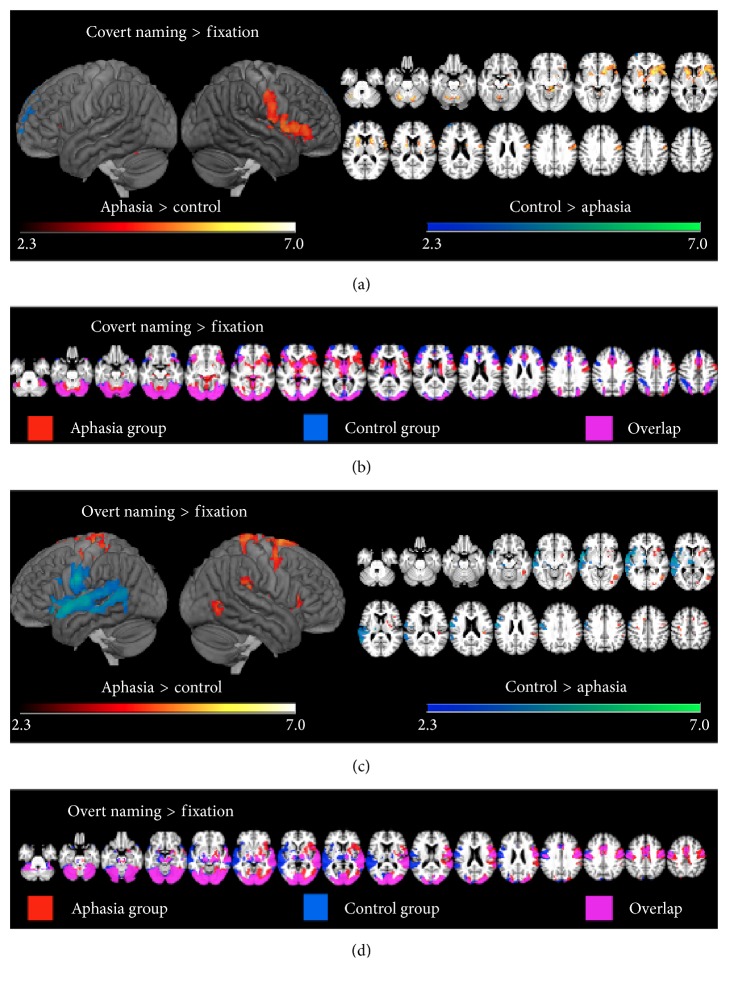
(a) Between-group contrasts showing activity during covert naming. (b) Within-group contrasts showing activity for both the aphasia and control groups during covert naming. (c) Between-group contrasts showing activity during overt naming. (d) Within-group contrasts showing activity for both the aphasia and control groups during overt naming.

In the covert > fixation contrast (Table 2, Figures 1(a)-1(b)), the aphasia group showed greater activity than the control group mostly in bilateral basal ganglia, bilateral cerebellum, but also the right ventral central sulcus and right IFG. The aphasia group underactivated the left frontal pole, cingulate cortex, and bilateral clusters in the superior frontal gyrus compared to controls. In within-group contrasts, both groups showed significant activation in the visual cortex. The control group showed bilateral activation in the inferior parietal sulcus (IPS), while the aphasia group only showed significant activation in the right IPS. Likewise, the control group showed activation in the bilateral insula and pars opercularis (POp), as well as left PTr, while the aphasia group only activated the right PTr.

During overt naming (Table 3, Figures 1(c)-1(d)), the aphasia group overactivated dorsal regions bilaterally, in particular bilateral central sulcus, as well as right insula, right angular gyrus, and right Heschl's gyrus. The aphasia group underactivated, relative to controls, the left IFG, insula, superior temporal gyrus (STG), and cerebellum. In the within-group contrasts, both the aphasia group and control group activated the right superior temporal sulcus (STS), STG, temporal pole, and central sulcus. Only the control group activated these regions in the left hemisphere, while only the aphasia group showed activity in the right IFG.

### 3.2. Effect of Lesion Size on Activity

**Figure 2:**
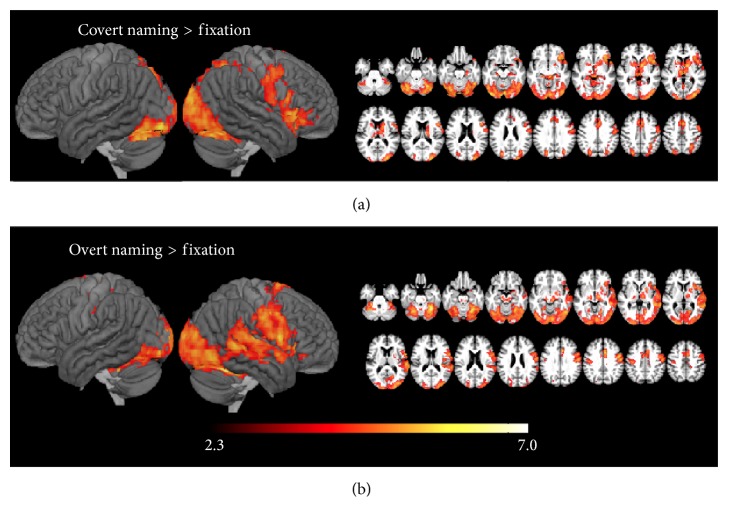
Regions where large left hemisphere lesions were related to greater activity during (a) covert naming and (b) overt naming.

Next, we looked for regions, within the aphasia group, where activity during covert and overt naming was predicted by large lesions (Table 4, Figure 2). During covert naming, larger lesion size predicted widespread right hemisphere activity, especially in the central sulcus, POp, and PTr, but also in bilateral visual cortex, cingulate, IPS, and basal ganglia. During overt naming, larger lesion size predicted activation in bilateral central sulcus, cingulate, and cerebellum, but activity was heavily right lateralized, especially in right PTr, posterior STS, and posterior STG.

We also looked for regions where activity was greater in participants with smaller lesions. There were no areas where activity was significantly predicted by smaller lesions at our threshold.

### 3.3. Lesions at ROIs and Behavior

Regions of interest were drawn based on peaks from the control group maps (Table 5). For covert naming, ROIs included the left and right IPS, left and right insula, and a left dorsal POp peak. Another peak in the left cingulate met requirements for an ROI, but only one participant in the aphasia group had a lesion in this area, so it was removed from further analysis. For overt naming, ROIs were selected in both the left and right motor cortex and STS.

We then tested whether lesion statuses in the left ROIs were related to naming performance in the scanner, using linear regression. Lesions in the left insula were related to worse naming, *t*(37) = −2.4, *P* < 0.05, but this effect did not hold when lesion size was introduced as a control variable, *P* > 0.30. Lesions in the left insula, dorsal POp, motor cortex, and STS had no relationship with naming performance in the scanner.

### 3.4. Effect of Lesion Location on Remapping and Whole-Brain Analysis

We then carried out whole-volume analysis using the aphasia group, testing whether lesion status at each left hemisphere ROI resulted in different patterns of activity in the rest of the brain, with lesion volume added as a nuisance variable. For covert naming, at a cluster corrected *P* < 0.01, there was no difference between people with left IPS lesions versus those without lesions at the IPS site.

**Figure 3:**
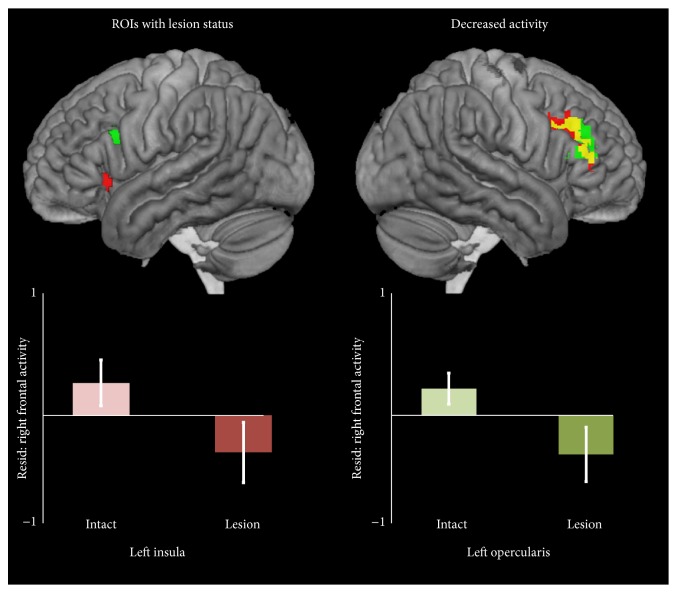
Lesions in the left insula and left opercularis were associated with less activity in the right middle frontal gyrus and right pars triangularis. Bar graphs show activity level in the right ROIs relative to the control sample, controlling for lesion volume.

People with left insula lesions showed less activity in the right middle and inferior frontal gyri, with peaks in the right middle frontal gyrus and right dorsal PTr. Participants with left POp lesions also showed less activation in nearly overlapping regions, including right dorsal POp (Figure 3, Table 6). The similarity of these results is likely due to the significant overlap between participants with left insula lesions and participants with left POp lesions.

We then took the cortical peak in this right hemisphere cluster, located in dorsal PTr, and extracted the activation levels for covert naming relative to baseline. The activation level in each participant with aphasia was transformed into a *z*-score centered on the control group mean. We carried out two tests, to examine whether activity in this area was related to PNT score, in the group with left POp lesions (*N* = 16) and in the group with intact left POp (*N* = 23), again controlling lesion size. There was no significant relationship between activity in the right peak activation and naming in either group (both *P*'s > 0.25). The same analyses were also done, dividing participants based on lesion status at left insula (intact *N* = 21, lesion *N* = 18), but again no relationship was found for either group (both *P*'s > 0.80).

No group differences in activity in the whole-volume analysis were identified based on lesion status at the two left hemisphere overt naming ROIs, left motor cortex and left STS, at this threshold.

### 3.5. Effect of Lesion Status in One ROI on Activity in Other ROIs

Finally, we tested whether lesion status in each left hemisphere ROI affected activity levels in all other, left and right hemisphere, ROIs derived from the healthy control sample, controlling for lesion size.

For covert naming, only one relationship was marginally significant. Participants with left insula lesions had marginally greater activity in the left POp, but this effect was unreliable at *P* = 0.07.

**Figure 4:**
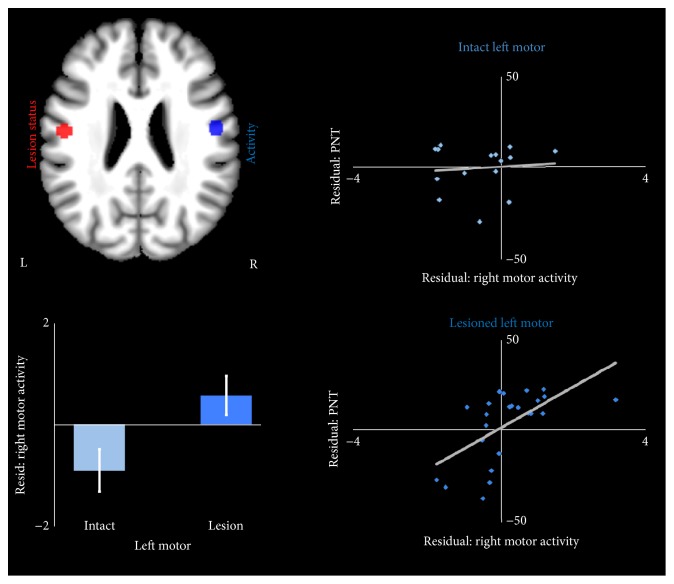
Lesions in the left motor cortex were associated with greater activity in the right motor cortex. The bar graph shows activity level in the right motor ROI relative to controls, controlling for lesion volume. Scatter plots show the relationship between activity and performance on the PNT, controlling for lesion volume, in the intact left motor group and in the group with lesions in left motor cortex.

For overt naming, lesions in the left motor cortex were related to significantly greater activity in the right motor cortex, *t*(36) = 2.91, *P* < 0.01, while lesions in the left STS were associated with lower activity in right motor cortex, *t*(36) = −2.24, *P* < 0.05 (Figure 4).

We then tested whether right motor cortex over- or underactivation predicted naming performance in people with and without left motor lesions. As with the IFG analysis above, we calculated *z*-scores for activity in participants with aphasia, centered on the control group mean. For participants in the aphasia group with intact left motor cortex (*N* = 15), there was no relationship between right motor activity and naming, *t*(12) = −0.28, *P* = 0.75. However, for participants with left motor lesions (*N* = 24), right motor activity was positively associated with naming, *t*(21) = 3.67, *P* = 0.001.

## 4. General Discussion

This study addressed the relationship between stroke distribution and naming activity in chronic left hemisphere stroke survivors. Specifically, we examined whether overall size of the lesion, and damage of different nodes in the normal naming network, results in different patterns of brain activity. The analysis approach allowed us to test several current hypotheses regarding poststroke plasticity in language networks. Overall, the results support the prevalent notion that larger strokes result in greater usage of right hemisphere areas and further demonstrate that damage to different left hemisphere language nodes results in different patterns of activity in surviving nodes. The specific results, however, present challenges for the interhemispheric inhibition model and suggest that other mechanisms of behavioral and biological plasticity might better account for the data.

### 4.1. Residual Left Hemisphere Language Activity and Perilesional Recruitment

A striking finding from the overall group analysis was a failure of people with aphasia to activate normal left hemisphere brain areas associated with speech production, including the ventral sensorimotor cortex and the superior temporal cortex [38], during overt naming. These areas were robustly activated by the control group but not the group with aphasia, a difference confirmed in the direct between-group comparison. This finding cannot be related to direct lesion damage to these areas, as lesioned voxels were excluded from the analysis on a person-by-person basis. Rather, this pattern suggests a failure to activate spared left hemisphere speech production areas due to lesions elsewhere in the network. This explanation was not clearly supported by the ROI analysis, however, in which lesions in left hemisphere overt naming nodes did not relate to decreased activity in other spared left hemisphere nodes. Of note, because the ROIs were based on peak locations of activity in the control group, they were all located in the grey matter. It is possible the decreased activity observed in normal left hemisphere speech areas was primarily driven by disconnection from other network nodes due to white matter damage, which was not examined here. Regardless, the failure to activate normal left hemisphere areas involved in naming begs the question of whether and where compensation might be occurring in spared brain areas.

One prominent idea regarding poststroke language network plasticity proposes that left hemisphere perilesional areas surrounding the stroke that were previously involved in other functions are recruited into the language network to compensate for loss of language nodes [8]. In our voxelwise analysis, we found only weak evidence for these effects, with small areas of increased activity relative to controls in left dorsal frontal cortex during overt naming. We note that the analysis may not have been sensitive to these effects due to variability in stroke distributions. In the more sensitive ROI analysis, we found that lesions in the left insula were associated with marginally higher covert naming activity levels in the left dorsal POp, but this effect was weak and difficult to interpret given how few of our participants had one of these nodes lesioned and the other intact. Overall, this study provides little evidence either for or against perilesional compensation.

### 4.2. Lesion Size and Right Hemisphere Recruitment

Another prominent mode of proposed reorganization after stroke is the recruitment of homotopic areas in the right hemisphere. It has frequently been suggested that overall lesion size in the left hemisphere may relate to right hemisphere recruitment [7, 28, 29]. This proposed relationship is sometimes based on the interhemispheric inhibition model, even though a relationship between lesion size and contralesional recruitment is not supported by animal models, in which small sensorimotor lesions result in an increase in synaptogenesis and astrocytic volume contralateral to the stroke, while large lesions result in decreases in both of these measures, likely due to denervation-induced atrophy [37]. Alternatively, the proposed relationship between lesion size and right hemisphere engagement is sometimes based on the logical argument that people with relatively small lesions have sufficient viable left hemisphere tissue to support language and may not require right hemisphere compensation, whereas people with large lesions have little viable left hemisphere tissue and must rely on the right hemisphere to a greater extent. Although a positive relationship between lesion size and right hemisphere recruitment is frequently discussed in the literature, there is surprisingly little direct empirical evidence of this relationship. One prior study examined the laterality indices of eight people with aphasia and found that large lesions were associated with greater right than left hemisphere activation during picture naming but not during a semantic decision task [17]. Here, we present strong support for this effect in both covert and overt naming in a large sample. Participants with larger lesions showed greater activity in right hemisphere areas homotopic to the normal left hemisphere language network. Notably, for overt naming, the specific pattern of activity related to lesion size in the right hemisphere (Figure 2(b)) closely mirrored the pattern of decreased activity relative to controls in the left hemisphere (Figure 1(c)).

The mechanisms underlying the increased activation of the right hemisphere in people with large lesions are unclear. This effect may not reflect plasticity at all but rather the increased effort required for language tasks by people with larger lesions. It is not unusual for more difficult tasks to elicit greater activity throughout the brain, including homotopic right hemisphere areas for language tasks [23]. Participants with large lesions likely exert greater effort in retrieving and producing the names of items and thus show overactivation during the task. Notably, activity in the bilateral visual cortex was also related to lesion size during both covert and overt naming here, suggesting that greater activity in general may be related to greater effort and longer looking times. Like other recent studies, we restricted our analysis to correct trials only in part to minimize these effort effects. However, even when producing a correct naming response, it is likely the people with large lesions may expend more effort than those with small lesions.

Similarly, it has recently been proposed that some right hemisphere overactivation observed in aphasia could be explained by recruitment of domain general attentional systems [39, 40] rather than language system reorganization per se. As above, the right hemisphere activity may similarly relate to the overall greater difficulty of language tasks for people with aphasia, although, under this hypothesis, the right hemisphere activity does not contribute to computations specific to language at all. Although this may explain part of the effect, we think it is unlikely to explain all right hemisphere overactivation in aphasia, given evidence here and elsewhere that activation of some right hemisphere nodes relates to the location of damage in the left and that the tasks that activate specific right hemisphere nodes are the same as those that activate the homotopic left hemisphere nodes in healthy controls [4].

Alternatively, explicit or implicit strategies used to compensate for deficits may result in recruitment of brain networks not used by healthy controls for language tasks, including the right hemisphere. As in the proposed overreliance on domain general systems, activity related to use of these strategies during scanning would not reflect any true plasticity in the language network. However, ultimately, reliance on domain general resources or alternate strategies in the long term could reinforce new neuronal connections and result in permanent neuroplastic changes in the network. In this case, differential activity could be observed even if the person does not actively use any alternate strategies during scanning. Melodic intonation therapy provides a clear example of an explicit compensatory strategy that can induce long lasting changes in network structure [41]. However, compensatory strategies need not be directly related to specific therapeutic experiences. For instance, a person who fails to retrieve the phonology of a word may attempt to visualize its spelling without any therapeutic training to do so. Changes in brain organization related to these type of strategic shifts may or may not relate to lesion size and location. For example, compensatory strategies involving pragmatic aspects of language or alternate forms of communication might be most used by people with large lesions and severe aphasia, potentially resulting in a relationship between lesion size and remodeling in brain areas involved in these functions. In the example of melodic intonation therapy, people with large frontal lesions causing nonfluent aphasias are most likely to receive this type of treatment [42], so this bias in exposure to intonation-based treatment could result in a relationship between left frontal strokes and right hemisphere changes that are behaviorally, rather than neurobiologically, driven. Spontaneous strategies such as mental visualization of word spellings could similarly relate to lesion location, assuming specific stroke distributions give rise to a pattern of deficits and preserved abilities that make these strategies advantageous. Importantly, however, reorganization related to strategic shifts should not necessarily occur in areas that are homotopic to the lesion.

### 4.3. Remapping in Homotopic Nodes

In contrast to the types of behaviorally driven plasticity described above, some neuroplasticity after stroke may occur as a direct biological result of the stroke itself. The interhemispheric inhibition model is the most prominent theory of biologically driven plasticity in the intact hemisphere after stroke. One prediction of this model is that remapping into the right hemisphere in aphasia should be homotopic to the lesion and not only driven by an overall lack of remaining left hemisphere tissue. A second predication is that as right hemisphere regions inhibit the remaining left hemisphere tissue, activity in the right should be associated with worse performance.

Contrary to this hypothesis, we found that lesions in the left POp and insula led to robustly decreased covert naming activity in the right PTr and in the MFG just dorsal to the POp, even when controlling for lesion volume. Furthermore, activity in this right frontal region was not related to naming ability. This finding also stands in contrast to a recent meta-analysis showing, across many studies using various language tasks, people with left IFG lesions were more likely to activate the right IFG [4]. There are several possible explanations for this discrepancy. First, the right hemisphere activity here is not perfectly homotopic with the lesioned nodes, although the difference in location, compared to the dorsal POp node, is very small. Second, we did not account for the proportion of the region affected by stroke. Greater right IFG activity might be more likely in people with a greater proportion of the left IFG damaged, while we treated all participants with lesions at the left insula (or POp) site as one group. However, a recent study using a semantic task found no relationship between proportion of intact left IFG and right IFG activity [43], and as noted above, the animal literature shows that larger lesions can be associated with atrophy of contralateral cortex, rather than enhanced plasticity [44], corresponding with our findings here. Second, our study controlled for total lesion volume, which is rarely done in fMRI studies of aphasia, particularly in case studies or case series with few participants. It is likely that increased right IFG activity in prior studies is related to large lesions that include the left IFG and not specifically to lesions in the left IFG themselves. In support of this explanation, Figure 2 demonstrates that increased total lesion volume is associated with increased right IFG activity, among other areas. Finally, our study excluded participants with severe anomia, who could not correctly name at least 10% of the trials in the scanner. Other studies involving more severe patients, especially those that do not isolate activity related exclusively to correct trials, might reasonably produce a different result in the IFG. Therefore, questions for future study include whether homotopic remapping in the inferior frontal lobes is more likely in people with more severe aphasia and is related primarily to erroneous responses, as has previously been suggested in a small case series [20].

We did find evidence for homotopic remapping in motor cortex, in that left motor cortex lesions led to significantly greater right motor cortex activation during overt naming, controlling for total lesion volume. However, in contrast to the predictions of the interhemispheric inhibition model, overactivation of right motor cortex was associated with better naming performance in people with left motor lesions. These results join a growing body of literature suggesting that the right hemisphere may play a largely compensatory role, even in chronic stages of aphasia. Evidence for this begins in neuropsychological case studies, which have identified people who had recovered at least partially from aphasia following left hemisphere stroke and then redeveloped aphasia after a later right hemisphere disruption, whether due to intracarotid amobarbital injection or a second stroke [45–48]. These studies suggest that the right hemisphere can, to some degree, take over the language functions that the left hemisphere can no longer perform. A recent structural study found that greater grey matter volume in the right temporoparietal cortex related to better language production outcomes in chronic aphasia [49]. Other neuroimaging studies have shown positive relationships between right hemisphere activation and various language outcomes, supporting right hemisphere compensation [50–52]. Based on performance decrements after inhibitory TMS, other studies have also suggested that right hemisphere areas may be involved to some degree in phonology and naming in healthy people [53, 54]. Damage to left hemisphere language nodes may thus result in increased reliance on these right hemisphere language processors [55]. However, this explanation cannot account for the effects observed here, since the activity in right motor cortex related to naming performance only in people with left motor cortex lesions. This pattern strongly suggests a true compensatory relationship in which the right hemisphere node “takes over” for the corresponding lesioned left hemisphere area and demonstrates that specific biological mechanisms of plasticity beyond the interhemispheric inhibition model must be considered.

### 4.4. Alternate Biological Mechanisms of Right Hemisphere Recruitment

If not interhemispheric inhibition, what biological mechanism might explain recruitment of a homotopic node in the right hemisphere? The first possibility is that the interhemispheric inhibition hypothesis may be partially correct. It is possible that right hemisphere homotopic recruitment does result from transcallosal disinhibition but that this overactivation does not significantly suppress the surviving left hemisphere tissue. Behaviorally important right-to-left inhibition may simply never occur in language systems, may be restricted to particular areas such as the PTr, or may occur only in the case of relatively small lesions with some nearly homotopic left hemisphere tissue remaining to be inhibited [7].

However, other biological mechanisms might better explain compensatory recruitment of homotopic areas in the spared hemisphere after stroke. It has previously been noted that right hemisphere activity is maximal several weeks after a stroke causing aphasia [17]. If this activity resulted from direct disinhibition, the right hemisphere activity should occur immediately after the stroke. This kind of immediate right hemisphere recruitment has been observed after TMS-induced transient lesions [55, 56], but not after stroke. The gradual development of right hemisphere activation after stroke instead suggests a slower process, possibly relying on structural plasticity rather than a direct electrophysiological effect.

We suggest that axonal collateral sprouting from surviving neurons may provide an alternative neurobiological mechanism to explain both perilesional left hemisphere and homotopic right hemisphere recruitment in aphasia. This model is based on the principle that if two neurons send axons to the same target, they compete for synapses at the target. If one neuron dies, its axon degenerates and the surviving neuron's axon sprouts new collaterals near the target to take over empty synapses. Axonal collateral sprouting is observed throughout the central and peripheral nervous systems, for instance, in the development of ocular dominance columns and the delineation of motor units at the neuromuscular junction. It is known to play a role in reorganization after spinal cord injury [57] and brain injury, including stroke [58]. Competition has been shown to guide axonal sprouting in the sensory-motor spinal circuits in adult rats [59], and unbalanced endogenous activity dramatically affects receptor targeting in tracts crossing the corpus callosum [60]. Although not the only mechanism that can explain “take over” of prior functions by new brain areas, axonal collateral sprouting has previously been demonstrated to account for this phenomenon. For example, when dorsal route fibers to the hippocampus are destroyed in rats, ventral route fibers take over these connections over a period of months resulting in ultimate recovery of innervation patterns [61]. Further, axonal collateral sprouting is altered by the experience of the organism [62], providing a specific biological mechanism for function and neuroanatomic changes induced by speech-language therapy in people with aphasia.

Under this hypothesis, spared brain regions that share axonal targets with the lesioned tissue are the most likely to be recruited after stroke. This suggests that areas engaged to compensate might be predictable based on coconnectivity patterns before the stroke. Neighboring neurons are likely to share axonal targets, thus providing a basis for perilesional recruitment in the case of relatively small lesions. In some cases, such as motor areas innervating proximal limb muscles and the tongue, the homotopic cortex in the intact hemisphere likely shares axonal projection targets with the lesioned neurons and can take over the lost synapses, resulting in functional recovery [63, 64].

Our findings were near the mouth areas of motor cortex, and this mechanism might explain our findings of right hemisphere mouth-area motor recruitment during overt but not covert naming, corresponding with better naming performance specifically when the left hemisphere mouth area of motor cortex is lesioned. However, motor cortex has also been shown in other neuroimaging studies to play a role in covert naming and prearticulatory processes [65]. Furthermore, Geranmayeh and colleagues have argued that, regardless of the role of motor cortex in healthy people, upregulation of the region in people with aphasia is a marker of increased demands on domain general systems such as cognitive control and attention [66], and the relationship between activity and recovery is more related to those processes than anything language specific. It is possible that the diverse, nonspecific role of the motor cortex in speech production may also prime it to be uniquely plastic and available for taking over cognitive function through axonal sprouting, when the homotopic region is lesioned.

Regardless of the specific role of each region, the broader pattern of right hemisphere recruitment may result from a cascading effect from one homotopic right hemisphere node taking over synapses from its left hemisphere counterpart at a shared axonal target. When this node takes over the function of the lesioned left hemisphere node, the entire right hemisphere network connected to this one node may become involved in compensation, at least to a degree, resulting in broad patterns of increased activity. Alternatively, homotopic areas of each hemisphere's association cortices may share common cortical and subcortical axonal projection targets, and synaptic competition may account directly for broad patterns of right hemisphere recruitment. Modern connectomics may help to test these ideas. If supported by additional data, this hypothesis may provide clear predictions regarding the availability and location of alternate processing nodes based on the specific anatomical structures damaged by an individual's stroke.

### 4.5. Limitations and Future Directions

One unexpected finding was that while both people with aphasia and controls showed widespread activity during both covert and overt naming, the differences between the two groups was more widespread during overt naming. Most strikingly, the temporal lobe, in particular the STS and STG, was not significantly active in either group during covert naming. The posterior STS and STG are involved in phonological processes, which can include phonological retrieval [67], verbal working memory [68], and a sensorimotor speech interface for speech productions [69], so it was expected to be active during the covert naming phase of the experiment. In our experiment, however, no response was collected during the covert naming phase. If a trial was answered incorrectly during overt naming, both the covert and overt naming phases were removed from analysis. But, we do not have any measurable evidence of what was occurring for each participant during the covert naming phase. It is possible that some participants were less engaged in the task and only really attempted to name the object when cued to make an overt response and thus did not activate articulatory regions during the covert naming phase. In general, the use of a low-level control condition in the fMRI task allowed us to identify activity for word retrieval and production broadly but prohibited a detailed accounting of the precise nature of the processing in any given area of activity.

In this study, we identified regions where plasticity was dependent on the site of the lesion in left frontal and motor tissue. However, it remains unclear whether these relationships are mediated by the degree to which critical areas for naming are damaged or preserved. The language network in the left hemisphere involves many regions which are critical for different aspects of language. The degree to which a critical area for naming is destroyed by the stroke may determine whether plasticity of naming function is even possible, regardless of the lesion status at other regions of interest such as motor or inferior frontal cortex. A goal of future research is to identify regions in which damage has a catastrophic effect on naming ability and then model how damage in these regions affects others in the system, both perilesional and homotopic.

## 5. Conclusions

In this study we tested three central hypotheses of the interhemispheric inhibition model. We found an overall greater rightward shift of activity dependent on lesion size. Taking this overall effect into account, specific patterns of right hemisphere plasticity depended on the specific location of the stroke. Furthermore, right motor activation was positively associated with naming ability but only in people with left motor lesions. This finding suggests that lesion site needs to be accounted for when considering both the cause of right hemisphere overactivation and the role of right hemisphere activity, in people with aphasia. It is unlikely that any single biological mechanism explains all aspects of poststroke reorganization, and as noted above, complex interactions between biology and the environment are expected. We suggest that future work on aphasia recovery should be guided by specific behavioral and biological hypotheses that lead to specific experimental predictions for brain imaging and stimulation studies. Interhemispheric inhibition is one such specific biological hypothesis, but it cannot account for the entire range of observed neuroplastic effects in aphasia. The field must begin to entertain equally specific alternate hypotheses, such as synaptic competition, in order to move forward.

## Supplementary Material

Supplementary Material 1: Heat map showing left hemisphere lesion distribution for all participants in the aphasia group.
Supplementary Material 2: Demographic information and performance WAB scores for each participant in the aphasia group.

## Figures and Tables

**Table 1:** Demographic information for the aphasia group and control group. Standard deviations are shown in parentheses. There were no differences between the groups on age, sex, education, or handedness.

	Aphasia group	Control group
Age (years)	59.8 (10.1)	58.7 (13.2)
Sex (male/female)	26/13	20/17
Education (years)	16.4 (2.8)	16.9 (2.6)
Handedness (right/left/ambidextrous/unknown)	33/4/0/4	33/3/1/0
Time since stroke (months)	52.9 (51.4)	—
WAB naming/word finding	7.1 (2.5)	—
WAB auditory-verbal comprehension	8.3 (1.5)	—
WAB repetition	7.0 (2.5)	—
WAB spontaneous speech	15.1 (4.9)	—

**Table 2:** MNI coordinates of activity in the covert naming > fixation contrast.

Group contrast	Peak *z*-value	*x*	*y*	*z*	Label
Aphasia > control	4.8	−22	16	8	Left basal ganglia
Aphasia > control	4.8	−20	−62	−28	Left lateral cerebellum
Aphasia > control	4.5	10	−40	−12	Right medial cerebellum
Aphasia > control	4.4	20	14	−2	Right basal ganglia
Aphasia > control	4.1	14	−62	−22	Right lateral cerebellum
Aphasia > control	4	−10	−28	0	Left thalamus
Aphasia > control	4.0	42	16	2	Right insula
Aphasia > control	4.0	65	10	0	Right ventral POp
Aphasia > control	3.9	62	−8	8	Right inferior central sulcus

Control > aphasia	3.9	−52	−40	60	Left IPS
Control > aphasia	3.7	−26	64	−2	Left frontal pole
Control > aphasia	3.7	26	54	26	Right superior frontal gyrus
Control > aphasia	3.5	−2	48	42	Left anterior cingulate cortex
Control > aphasia	3.1	−28	44	32	Left superior frontal gyrus

Aphasia group	8.1	36	90	0	Right visual cortex
Aphasia group	7.6	8	−84	−18	Right medial cerebellum
Aphasia group	6.8	10	0	6	Right thalamus
Aphasia group	6.6	−4	16	42	Left cingulate cortex
Aphasia group	6.2	50	20	−6	Right PTr
Aphasia group	6.2	−30	−92	4	Left visual cortex
Aphasia group	6.0	4	−34	−4	Right brainstem
Aphasia group	5.9	0	−22	6	Medial thalamus
Aphasia group	5.6	48	8	22	Right central sulcus
Aphasia group	5.2	30	−56	50	Right IPS
Aphasia group	5.2	−44	30	14	Left POp
Aphasia group	5.1	−16	6	6	Left thalamus
Aphasia group	5.1	50	18	−16	Right anterior STS
Aphasia group	5.0	18	−32	−8	Right posterior hippocampus

Control group	8.9	32	−92	12	Left visual cortex
Control group	7.2	0	28	36	Medial cingulate cortex
Control group	6.9	30	−66	54	Right dorsal IPS
Control group	6.6	−30	−60	48	Left dorsal IPS
Control group	6.6	38	22	−6	Right insula
Control group	6.5	−36	20	−4	Left insula
Control group	6.4	−50	28	24	Left dorsal POp
Control group	6.3	4	−36	−4	Right brainstem
Control group	6.3	54	16	−16	Right anterior STS
Control group	6.1	−48	12	24	Left ventral POp
Control group	6.0	48	10	24	Right central sulcus
Control group	6.0	54	38	12	Right POrb
Control group	5.9	−46	32	12	Left PTr
Control group	5.8	−32	56	14	Left frontal pole
Control group	5.7	28	−70	34	Right ventral IPS
Control group	5.5	−36	−50	44	Left ventral IPS

**Table 3:** MNI coordinates of activity in the overt naming > fixation contrast.

Group contrast	Peak *z*-value	*x*	*y*	*z*	Label
Aphasia > control	4.7	−28	−22	48	Left dorsal central sulcus
Aphasia > control	4.6	44	−66	0	Right visual cortex
Aphasia > control	4.6	24	0	66	Right dorsal central sulcus
Aphasia > control	4.0	−12	−26	64	Left middle cingulate cortex
Aphasia > control	4.0	−38	−10	48	Left central gyrus
Aphasia > control	3.9	40	−6	52	Right central gyrus
Aphasia > control	3.9	−14	10	42	Left anterior cingulate cortex
Aphasia > control	3.9	28	16	2	Right insula
Aphasia > control	3.8	40	−30	18	Right Heschl's gyrus
Aphasia > control	3.4	48	−34	24	Right angular gyrus
Aphasia > control	3.2	−32	−38	46	Left ventral IPS

Control > aphasia	5.2	−62	−4	−4	Left temporal pole
Control > aphasia	5.1	−62	−2	24	Left middle central sulcus
Control > aphasia	4.6	−10	−18	6	Left thalamus
Control > aphasia	4.5	−66	−40	4	Left posterior STS
Control > aphasia	4.1	−40	−14	−12	Left posterior insula
Control > aphasia	4.1	−4	−24	0	Left brainstem
Control > aphasia	4.0	−46	−30	6	Left posterior STG
Control > aphasia	3.9	−44	18	−6	Left PTr
Control > aphasia	3.9	−68	−30	8	Left middle STG
Control > aphasia	3.6	−16	−30	−16	Left hippocampus
Control > aphasia	3.4	−40	−82	−22	Left cerebellum

Aphasia group	7.7	54	4	36	Right central gyrus
Aphasia group	7.4	28	−80	−20	Right visual cortex
Aphasia group	7.4	6	6	52	Right cingulate cortex
Aphasia group	6.4	66	−22	−2	Right middle STG
Aphasia group	6.1	48	−34	2	Right posterior STS
Aphasia group	5.9	−46	−16	36	Left central sulcus
Aphasia group	5.6	12	−16	0	Right thalamus
Aphasia group	5.4	44	10	−6	Right PTr
Aphasia group	5.4	36	20	2	Right anterior insula
Aphasia group	5.4	56	−32	8	Right posterior STG
Aphasia group	5.3	−26	−94	12	Left visual cortex
Aphasia group	5.3	64	−40	22	Right angular gyrus
Aphasia group	5.1	40	−12	14	Right posterior insula
Aphasia group	5.0	−8	8	40	Left middle cingulate cortex
Aphasia group	5.0	38	−30	18	Right Heschl's gyrus
Aphasia group	4.9	32	−14	−10	Right middle hippocampus
Aphasia group	4.5	22	10	0	Right basal ganglia
Aphasia group	3.3	2	−54	−26	Right medial cerebellum
Aphasia group	3.3	−34	−20	48	Left central gyrus
Aphasia group	3.2	10	−28	−24	Right brainstem

Control group	8.5	−10	−104	−2	Left visual cortex
Control group	7.8	−50	−12	26	Left central sulcus
Control group	6.5	−16	−28	−12	Left middle hippocampus
Control group	6.5	22	−28	−10	Right middle hippocampus
Control group	6.4	68	−28	2	Right posterior STS
Control group	6.2	14	−18	2	Right basal ganglia
Control group	6.2	−64	−42	6	Left posterior STS
Control group	6.1	−2	2	58	Left middle cingulate cortex
Control group	6.0	−66	−2	−6	Left temporal pole
Control group	5.8	58	14	−16	Right temporal pole
Control group	5.6	−40	−30	8	Left Heschl's gyrus
Control group	5.3	66	−6	10	Right MTG
Control group	5.0	14	−24	−12	Right brainstem
Control group	4.1	−32	24	2	Left anterior insula
Control group	3.8	−46	8	22	Left dorsal POp
Control group	3.6	−36	4	2	Left middle insula

**Table 4:** MNI coordinates of activity associated with large lesions, for both the covert naming and overt naming contrasts.

Task	Peak *z*-value	*x*	*y*	*z*	Label
Covert > fixation	5.5	4	−82	−16	Right visual cortex
Covert > fixation	5.4	34	−64	−24	Right cerebellum
Covert > fixation	5.3	−40	−88	−14	Left visual cortex
Covert > fixation	5.3	6	−32	−8	Right brainstem
Covert > fixation	5.1	12	0	2	Right basal ganglia
Covert > fixation	4.9	−26	−76	40	Left ventral IPS
Covert > fixation	4.8	30	−56	50	Right IPS
Covert > fixation	4.7	4	6	56	Right cingulate cortex
Covert > fixation	4.3	66	0	10	Left central gyrus
Covert > fixation	4.1	−18	8	4	Left basal ganglia
Covert > fixation	3.8	−24	−64	56	Left dorsal IPS

Overt > fixation	6.6	32	−58	−24	Right cerebellum
Overt > fixation	6.1	−26	−64	−28	Left cerebellum
Overt > fixation	5.4	4	4	52	Right cingulate cortex
Overt > fixation	5.4	68	−18	0	Right middle STS
Overt > fixation	5.3	66	2	10	Right PTr
Overt > fixation	5.1	52	−38	−6	Right MTG
Overt > fixation	4.3	−46	−16	36	Left central sulcus
Overt > fixation	4.3	22	8	0	Right basal ganglia
Overt > fixation	4.0	38	−12	14	Right posterior insula
Overt > fixation	3.9	−14	−34	54	Left cingulate cortex
Overt > fixation	3.8	−20	−20	0	Left external capsule
Overt > fixation	3.4	32	−14	−12	Right hippocampus

**Table 5:** Regions of interest drawn from the contrasts in the control group.

Contrast	Control group peak	*x*	*y*	*z*	Label	Subjects in aphasia group with lesions at ROI (out of 39 in total)
Covert > fixation	6.61	−30	−60	48	Left IPS	11
Covert > fixation	6.48	−36	20	−4	Left insula	18
Covert > fixation	6.38	−50	28	24	Left dorsal POp	16
Covert > fixation	6.91	30	−66	54	Right IPS	—
Covert > fixation	6.56	38	22	−8	Right insula	—

Overt > fixation	7.77	−50	−12	26	Left motor cortex/central sulcus	24
Overt > fixation	6.18	−64	−42	6	Left posterior STS	20
Overt > fixation	7.12	52	−10	26	Right motor Cortex/central sulcus	—
Overt > fixation	6.4	68	−28	2	Right posterior STS	—

**Table 6:** Areas where decreased activity was related to lesions in the left frontal lobe ROIs.

Lesion status ROI	Peak *z*-value	*x*	*y*	*z*	Label
Insula	−3.8	30	10	18	Right subcortical IFG
Insula	−3.6	46	22	32	Right superior frontal gyrus
Insula	−3.6	44	30	14	Right dorsal POp

Opercularis	−3.8	42	26	16	Right dorsal POp
Opercularis	−3.7	30	10	18	Right subcortical IFG
Opercularis	−3.2	48	26	30	Right superior frontal gyrus
